# Genome Sequence of Rhizobacterium *Serratia marcescens* Strain 90-166, Which Triggers Induced Systemic Resistance and Plant Growth Promotion

**DOI:** 10.1128/genomeA.00667-15

**Published:** 2015-06-18

**Authors:** Haeyoung Jeong, Joseph W. Kloepper, Choong-Min Ryu

**Affiliations:** a Super-Bacteria Research Center, Korea Research Institute of Bioscience and Biotechnology (KRIBB), Daejeon, Republic of Korea; b Biosystems and Bioengineering Program, University of Science and Technology (UST), Daejeon, Republic of Korea; c Department of Entomology and Plant Pathology, Auburn University, Auburn, Alabama, USA

## Abstract

The rhizobacterium *Serratia marcescens* strain 90-166 elicits induced systemic resistance against plant pathogens and herbivores and promotes plant growth under greenhouse and field conditions. Strain 90-166 secretes volatile compounds, siderophores, salicylic acid, and quorum-sensing autoinducers as bacterial determinants toward plant health. Herein, we present its draft genome sequence.

## GENOME ANNOUNCEMENT

The main mechanisms of plant growth-promoting rhizobacteria (PGPR) have been ascribed to molecular plant-bacteria interactions, such as phosphate solubilization, production of antimicrobial compounds, and plant growth hormones. In addition, some PGPR strains are known to achieve biological control of plant diseases by induced systemic resistance (ISR) (1). For example, *Serratia marcescens* strain 90-166, isolated in 1990 from a field-grown plant in Alabama, USA (initially misidentified as *S. plymuthica*), and originally selected for biological control capacity against *Rhizoctonia solani* on cotton (2), can elicit ISR against cucumber mosaic virus (3), *Colletotrichum orbiculare* (4), *Erwinia tracheiphila* (5), *Pseudomonas syringae* pv. lachrymans (6), and *Fusarium oxysporum* (7). It has been suggested that *S. marcescens* 90-166 mediates ISR by a quorum-sensing-dependent mechanism (8).

The genome of strain 90-166 was sequenced using the Illumina HiSeq 2000 platform by the National Instrumentation Center for Environmental Management from Seoul National University (Seoul, Republic of Korea). From a genomic library of an average insert size of 350 bp, 31,155,434 paired reads (101 cycle, approximately 570-fold coverage) were produced. Quality trimming, filtration by length, and *de novo* assembly was carried out using the CLC Genomics Workbench 8.0 (CLC bio). The assembly consists of 63 contigs and a total length of 5,484,396 bp. The maximum contig length and *N*_50_ were 897,417 bp and 632,250 bp, respectively. Automatic genome annotation was carried out using the NCBI Prokaryotic Genome Annotation Pipeline (PGAP) service and the RAST server (9). Average nucleotide identity (ANI) analysis with publicly available complete genome sequences of *Serratia* genus and with the draft genome sequence of *Serratia marcescens* type strain ATCC 13880 species (10) using the JSpecies software (11) revealed that strain 90-166 is most similar to *S. marcescens* strains WW4 (ANI value, 95.6%) (12), ATCC 13880 (ANI value, 95.3%), and Db11 (ANI value, 95.3%). The closest neighbor suggested by the RAST analysis was *S. marcescens* Db11.

From the genome annotation, we identified 2,3-butanediol dehydrogenase (EC 1.1.1.4) corresponding to production of 2,3-butanediol (8) that has been previously reported as volatile-mediated plant growth and ISR, isochorismate synthase (EC 5.4.4.2) for a siderophore, enterobactin (13), and *N*-acyl-l-homoserine lactone synthetase for secretion of a bacterial quorum-sensing signal homoserine lactone (8). More intriguingly, the genome of strain 90-166 contains genes encoding 6-methyl salicylic acid synthase for salicylic acid secretion and a LysR-family transcriptional regulator BsdA, an activator of BsdBCD in response to salicylic acid, indicating that strain 90-166 produces a plant defense hormone salicylic acid and responses salicylic acid that originates in bacteria and plants as a host of strain 90-166.

### **Nucleotide sequence accession number**s.

This whole-genome shotgun project has been deposited at DDBJ/EMBL/GenBank under the accession no. LCWI00000000. The version described in this paper is version LCWI01000000.
